# Sequential transcatheter arterial chemoembolization, three-dimensional conformal radiotherapy, and high-intensity focused ultrasound treatment for unresectable hepatocellular carcinoma patients

**DOI:** 10.7555/JBR.26.20120016

**Published:** 2012-04-10

**Authors:** Shengfa Ni, Lingxiang Liu, Yongqian Shu

**Affiliations:** Department of Oncology, the First Affiliated Hospital, Nanjing Medical University, Nanjing, Jiangsu 210029, China.

**Keywords:** transcatheter arterial chemoembolization (TACE), three-dimensional con-formal radiotherapy (3-DCRT), high-intensity focused ultrasound (HIFU), hepatocellular carcinoma

## Abstract

The purpose of this study was to evaluate the outcome of patients with unresectable hepatocellular carcinoma (HCC) treated by sequential therapy of transcatheter arterial chemoembolization (TACE), three-dimensional conformal radiotherapy (3-DCRT) and high-intensity focused ultrasound (HIFU). From October, 2005 to September, 2010, 120 patients with unresectable HCC received the sequential treatments of several courses of TACE followed in 2-4 weeks by 3-DCRT and then a single session of HIFU with a curative intent. The median tumor irradiation dose was 40 Gy. Tumor response, toxicity and overall survival rate were analyzed. Clinicopathologic factors affecting the primary technique effectiveness and overall survival rates were investigated by univariate analysis or multivariate analysis. All 120 HCC patients were followed up by the last follow-up time. Among these patients, hepatic toxicities due to treatment were notable in 9 cases. Gastrointestinal bleeding after the overall treatment occurred in 2 cases, leukopenia of grade III was detected in 1 case, radiation-induced liver disease (RILD) was observed in 2 patients, and first- and second-degree skin burn around the HIFU treatment zone were observed in 2 patients and 1 patient, respectively. Among 120 patients, 23, 83 and 14 cases achieved partial response, stable disease and progressive disease, respectively. The overall survival rates at 1 year, 3 years and 5 years were 70%, 35% and 15%, respectively, with a median survival time of 26 months. Both Child-Pugh liver function grading and radiation dose were determined to be independent predictors for overall survival revealed by the multivariate analysis. It is concluded that the sequential therapy of TACE, 3-DCRT and HIFU is a promising therapeutic regimen for unresectable HCC.

## INTRODUCTION

Hepatocellular carcinoma (HCC) is the most common type of primary liver cancer, with a high prevalence in Asia and an increasing incidence in Western countries[1]. For patients with early-stage HCC, surgical resection and liver transplantation are effective treatments. The 5-year survival rate after surgical resection is approximately 40%-70%, whereas ranging from 60% to 70% after transplantation[2]. Most patients with HCC, however, are diagnosed at an intermediate or advanced stage when few effective therapies are available. Transcatheter arterial chemoembolization (TACE) is considered as a meaningful adjuvant treatment for technically unresectable HCC. Unfortunately, TACE is less effective for patients with larger sized tumors[3]. Conventional external irradiation has been used effectively to complement palliative treatment of unresectable HCC, but its use has been limited by the risk of hepatic failure due to irradiation. Fortunately, three-dimensional conformal radiotherapy (3-DCRT) cannot only maximize the radiation dose to the hepatic tumor but also minimize liver injury[4]. With advancement in technologies, local ablation therapies have emerged as effective treatment options. These include cryoablation therapy, interstitial laser therapy, microwave coagulation, radiofrequency ablation (RFA), and high-intensity focused ultrasound (HIFU)[1]. Among these treatment options, HIFU is the only treatment modality that is completely extracorporeal. Because high-intensity energy is focused on a small volume, damage to tissues between the transducer and the target lesion is minimized. The mechanical effect involves cavitation, microstreaming, and radiation forces. With these destructive mechanisms, irreversible cell death occurs through coagulative necrosis and apoptosis. In this clinical study, we evaluated the safety and efficacy of sequential therapy of TACE, 3-DCRT, and HIFU in 120 HCC patients.

## SUBJECTS AND METHODS

### Study subjects

We retrospectively reviewed the clinicopathological data of HCC patients who sought surgical treatment at the Department of Surgery, the First Affiliated Hospital of Nanjing Medical University, Nanjing, China between October, 2005 and September, 2010. HCC was diagnosed based on radiological features shown by computed tomography (CT) scan or magnetic resonance imaging (MRI) scan and/or increased serum alpha-fetoprotein (AFP) concentrations (over 400 µg/L). Patients were included in the study if 1) they had inoperable HCC at an intermediate to advanced stage due to liver cirrhosis or cardiovascular diseases; 2) if they had Child-Pugh liver function class A or B; 3) if their Eastern Cooperative Oncology Group Scale of Performance Status (ECOG PS) was 0-1; 4) if they had a life expectancy of at least 3 months; 6) if they had complete follow-up data. Patients with advanced diseases due to tumor invasion to major intrahepatic blood vessels or extrahepatic metastasis were excluded from the study[5]. During the study period, 120 patients with unresectable HCC received sequential therapy of TACE, 3-DCRT and HIFU. All results were based on the treated population (***Table 1***). In 3 patients with Child-Pugh grade B, the maximum dimensions of tumor were 6.5, 7.2 and 10.6 cm, respectively.

**Table 1:** Baseline demographic and disease characteristics of hepatocellular carcinoma patients

Characteristics	Patients (*n* = 120)
Median age, years (range)	52 (32-78)
Gender	
Male	105
Female	15
ECOG PS	
0	10
1	110
Child-Pugh status	
A	117
B	3
AFP (µg/L)	
< 400	75
> 400	45
HBV	
Positive	81
Negative	39
Tumor size (cm)*	
Median (range)	7.8 (5-18)
BCLC stage	
B	90
C	30
TACE times	
1-2	95
3	25
Radiation dose	
< 45Gy	62
> 45Gy	58
HIFU times	
1-2	82
3	38

BCLC: Barcelona Clinic Liver Cancer; HBV: hepatitis B virus; HIFU: high-intensity focused ultrasound; TACE: transcatheter arterial chemoembolization; ECOG PS: the Eastern Cooperative Oncology Group Performance Status.

*Tumor size: the maximum dimension of tumor.

### TACE procedure

TACE was performed using the Seldinger technique. Guided by arterial angiography, the tip of a 4F catheter was introduced into the tumor-feeding artery. In some cases, a microcatheter with 3F outer diameter was utilized for selective catheterization into the tumor-feeding artery. Oxaliplatin (75 mg/m^2^) was then infused through the catheter followed by a mixture of iodized oil (Lipiodol Ultrafluide, Guerbet, France) and epirubicin (60 mg/m^2^). Occasionally, gelatin sponge pledgets were used to enhance the embolic effect. If arterioportal shunts were detected on angiography, the gelatin foam particles or strips would be used to block the arteriovenous shunt. After TACE, postembolization syndrome was treated symptomatically. To prevent postembolization hepatic failure, liver-protection drugs such as reduced glutathione and diammonium glycyrrhizinate would be used. For the entire group, a median of 2 courses of TACE (range 1-6) was administered at an interval of about 46 weeks between treatments.

### 3-DCRT procedure

Approximately 2-4 weeks after the final TACE course, 3-DCRT was performed. A CT scan for treatment planning was first performed. All patients were immobilized using a vacuum-lock cradle and were trained to breathe as shallowly as possible. For patients whose tumor margins were not clear on plain CT, contrast CT scan or MRI were carried out to clarify the gross tumor volume (GTV) margin. By combining diagnostic CT or MRI with lipiodol deposit from TACE, the GTV could be delineated accurately. The liver, kidneys, stomach, duodenum and spinal cord were regarded as organs at risk (OAR). Planning target volume (PTV) was determined by adding 1.0–1.5 cm to the GTV. Due to respiration, the tumor's motion was also taken into account when deciding PTV. Aided by the beam's eye view, 4-6 coplanar or non-coplanar fields were designed. Cumulative dose-volume histogram (DVH) was used to evaluate each treatment plan. The optimization of the 3-DCRT plan was performed according to our Radiation Department's previous experience. Irradiation was delivered by 6-mega volt (MV) X-ray. The mean dose to normal liver (MDTNL) (normal liver volume = total liver volume - PTV) was limited to 30 Gy. Manipulation of dose schedule by conventional fractionation (2 Gy/fraction and five fractions per week) allowed minimization of MDTNL. The median tumor irradiation dose was 40 Gy (range 24-60 Gy). The overall treatment time ranged from 2 to 10 months.

### HIFU treatment procedure

About 2 weeks after 3-DCRT, HIFU was performed. The JC HIFU system (Chongqing Haifu Technology, Chongqing, China) was used in this study. The ablation process was guided by real-time ultrasound imaging. This system is composed of a real-time diagnostic ultrasound device, an integrated ultrasound therapy transducer (12 cm in diameter), a 6-directional therapeutic planning system, an ultrasound generator, a degassed water circulation unit, and a computer unit for automated master control. The focused ultrasound was produced by the transducer operating at 0.8 MHz (aperture 120 mm, focal length 150 mm). The target lesion was identified using a central 3.5-MHz diagnostic ultrasound probe, which was integrated in the center of the therapeutic transducer. Both diagnostic and therapeutic ultrasound beams were emitted simultaneously in the same direction. HIFU treatment was performed under general anesthesia to alleviate deep visceral pain caused by HIFU and to ensure immobilization of patients. Temporary inspiratory or expiratory control by the anesthesiologist helped to minimize liver movement caused by ventilation during the treatment. In selected patients with a tumor at the dome of the liver, artificial right pleural effusion was induced before HIFU treatment. Detailed planning was carried out according to the tumor size and location as detected by the diagnostic ultrasound transducer. Parallel slides of the target tumor with 5-mm separation were obtained. Using provisional therapeutic parameters based on the depth and vascular supply of the target tumor, tissue of each tumor slide was completely ablated from deep to superficial region by successful sweeps of the HIFU head. The ablation process was repeated slide by slide to achieve entire tumor ablation. During the ablation process, gray-scale changes were noted in the ablation zone, signifying the effectiveness of ablation. Clinical details of all 120 patients were prospectively collected in a database. Clinical parameters included patient demographics, tumor characteristics, and treatment parameters (total treatment duration and acoustic power). Short-term outcome measures were post-HIFU complication rate, hospital mortality, and tumor responses. A complication was defined as any adverse event after HIFU, and hospital mortality was defined as any death in the same admission for the procedure. Tumor responses were classified as the primary technique effectiveness rate and secondary technique effectiveness rate, according to the recommendation by the International Working Group on Image-guided Tumor Ablation6. The primary technique effectiveness rate was defined as the percentage of tumors that were successfully eradicated following the initial course of HIFU, whereas the secondary technique effectiveness rate was defined as the percentage of tumors that have undergone successful repeated ablations following identification of local tumor progression. Tumor response to HIFU was assessed by MRI, which was performed 1 month after the procedure. Successful tumor ablation was defined as complete absence of hyperintensity signal in T2W images and absence of contrast enhancement within the original tumor region. Any contrast-enhancing area within the original tumor region on post-ablation MRI scan indicated a residual tumor. All patients had monitoring of serum AFP concentration, chest radiograph, and MRI scan every 3 months to detect tumor recurrence.

### Clinical assessment

During TACE, 3-DCRT or HIFU, patients were monitored weekly by physical examination, complete blood cell counts, and liver function tests. Approximately one month after completion of HIFU, patients were evaluated by physical examination, blood chemistry analysis and plain CT. Follow-up was performed monthly by means of telephone interviews with patients one month after HIFU. Tumor response was assessed by the Response Evaluation Criteria in Solid Tumors version 1.1 (RECIST v1.1). Acute toxicities associated with combined treatment were graded by using the National Cancer Institute Common Toxicity Criteria version 3.0 (NCI-CTCAE v3.0).

### Statistical analysis

The survival was estimated from the date of initiation of TACE with the Kaplan-Meier method. To assess the prognostic factors, univariate analyses and multivariate analyses by Cox proportional regression model were performed. The difference of survival was assessed by the log-rank test. All statistical analyses were performed using SPSS 18.0 (SPSS Inc., Chicago, IL, USA) at a 5% level of significance.

## RESULTS

### Follow-up

Follow-up was performed one month after HIFU. All patients had been followed up by the last follow-up time, which was completed in July, 2010. For the entire group, the median follow-up time was 14 months (range 2-80 months). At the last follow-up, 18 patients remained alive. Among the surviving patients, two patients received surgical resection after HIFU. By the last follow-up, 102 patients died. Among the deceased patients, one patient died of hepatic failure immediately after surgery for HCC, and two died of hepatic failure caused by radiation-induced liver disease (RILD). The primary cause of death in the remaining patients was disease progression.

### Tolerance and toxicity

During irradiation, nine patients were unable to tolerate a full course of treatment due to acute hepatic toxicity in 5 cases and worsening general condition in 4 cases. Treatment-related toxicity was assessed in all 120 patients according to NCI-CTCAE v3.0. Acute hepatic toxicities were notable in 15 patients with grade I in 8 cases, grade II in 1 case, and grade III in 6 cases, but all patients eventually recovered. Acute gastrointestinal complications of grade I occurred in 7 patients, while acute gastrointestinal bleeding occurred in 3 cases. Leukopenia of grade I was seen in 32 patients, grade II in 17 cases, and grade III in 1 case. RILD was observed in 3 patients over 3 months following 3-DCRT. These patients all were positive for hepatitis B virus (HBV) and had liver cirrhosis, but their liver function was Child-Pugh A. Their MDTNLs were 23.3, 23.6 and 24.9 Gy, respectively. Two of these patients died of hepatic failure in spite of active medical treatment. One patient who had RILD was still alive at the last follow-up but had worsened general condition and decreased hepatic function. First-degree and second-degree skin burn around the HIFU treatment zone was observed in 2 patients and 1 patient, respectively.

### Tumor response

For each patient, immediate tumor response was evaluated one month after HIFU. Among 120 patients, complete response (CR) was not observed, while we observed partial response (PR) in 23 patients, stable disease (SD) in 83 patients, and progressive disease (PD) in 14 patients. Among the 14 patients with PD, 10 patients had local tumor progression without distant metastasis, one had lung metastasis without local progression, one had bone metastasis without local progression, one had both lung metastasis and local progression, and one had both lung and bone metastasis.

### Survival rates

**Fig. 1:**
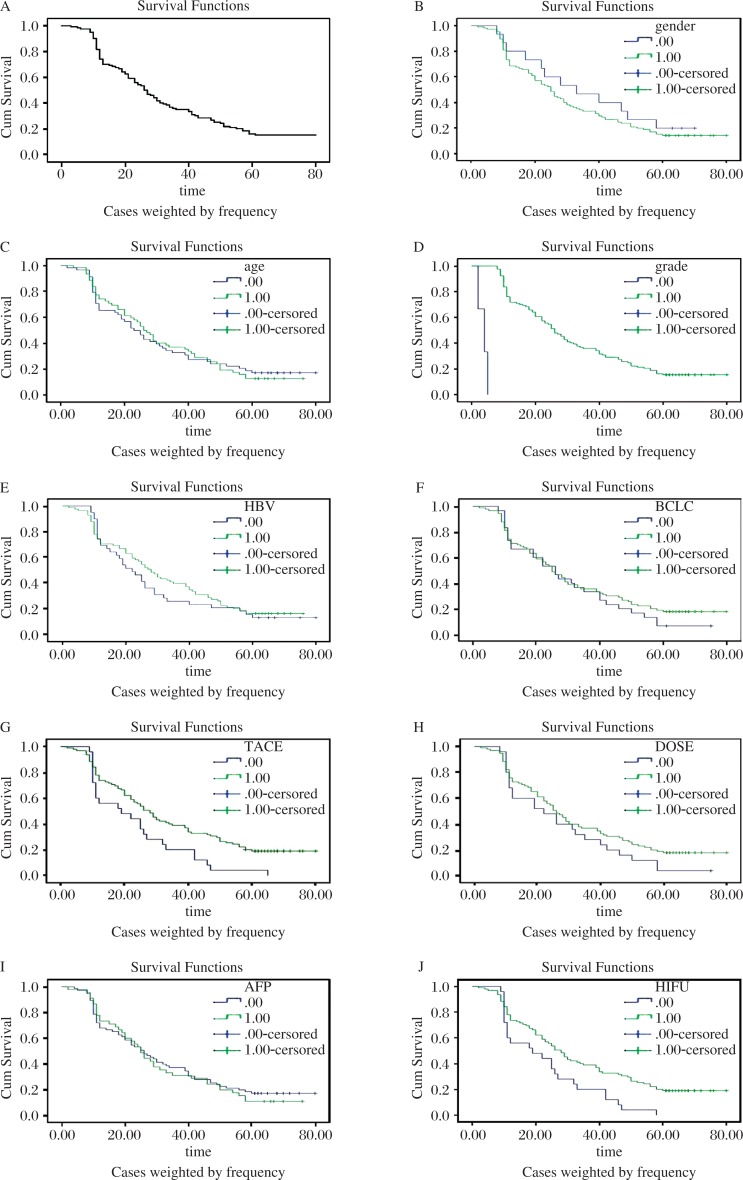
Kaplan-Meier survival estimates of 120 hepatocellular carcinoma (HCC) patients. A: The overall survival rates of 120 HCC patients treated by sequential therapy of TACE, 3-DCRT and HIFU. The effect on various prognostic factors including gender (B), age (C), Child-Pugh grade (D), HBV (E), Barcelona Clinic Liver Cancer (BCLC) stage (F), TACE times (G), radiation dose (H), alpha-fetoprotein (AFP) (I), and HIFU times (J) on survival.

The survival curve for patients is shown in ***Fig. 1A***. Cumulative survival rates at 1 year, 3 years, and 5 years were 70%, 35%, and 15%, respectively. The median survival time was 26 months (range 2-80 months), and the time to progression was 9.5 months (range 1-56 months).

### Prognostic factors affecting survival

Prognostic factors including age, gender, Child-Pugh status, serum AFP concentration, HBV, GTV, Barcelona Clinic Liver Cancer (BCLC) stage, portal vein thrombosis, TACE times, radiation dose and HIFU times were evaluated by univariate analysis. The analysis showed that age, gender, serum AFP concentration, HBV, and GTV had no significant effect on the overall survival (all *P* values > 0.05). However, Child-Pugh grade A, BCLC stage B, no portal vein thrombosis, TACE times of > 2, radiation dose of > 40 Gy and HIFU times of > 2 could predict a better overall survival (***Table 2***, ***Fig. 1B*** to ***1J***). The Cox proportional regression analysis revealed that both Child-Pugh grade and radiation dose were demonstrated as independent predictors for overall survival. Patients with Child-Pugh grade A and radiation dose of > 40 Gy had longer survival (***Table 3***).

**Table 2:** Univariate analysis of prognostic predictors for survival rate of hepatocellular carcinoma patients

Predictor	Number	Overall survival (%)			Median survival time (months)	Log-rank test	
		1 year	3 year	5 year		χ^2^ value	*P* value
Age(year)						1.130	0.288
< 52	62	70	35	11	15		
>52	58	62	35	16	19		
Gender						1.304	0.254
Male	105	70	36	15	19		
Female	15	67	33	20	16		
Child-Pugh grade						29.789	0.000
A	117	69	31	15	19		
B	3	0	0	0	5		
AFP(IU/ml)						0.899	0.638
< 400	75	74	58	38	44		
> 400	45	80	67	40	47		
HBV						0.128	0.720
Positive	81	62	28	13	19		
Negative	39	72	31	14	17		
PTV (cm^3^)						0.494	0.482
> 125	25	68	63	25	44		
< 125	95	83	55	41	47		
BCLC stage						9.661	0.002
B	90	72	38	17	23		
C	30	50	9	9	14		
TACE times						6.035	0.014
1-2	95	70	25	6	18		
>3	25	68	61	46	44		
Radiation dose						13.618	0.000
> 40Gy	62	77	42	22	27		
< 40Gy	58	52	19	9	13		
HIFU times						6.035	0.014
1-2	38	70	25	6	18		
> 3	82	68	61	46	44		

AFP: Alpha-fetoprotein; HBV: hepatitis B virus; BCLC: Barcelona Clinic Liver Cancer; TACE: transcatheter arterial chemoembolization; PTV: planning target volume; HIFU: high-intensity focused ultrasound.

(*n* = 120)

## DISCUSSION

For patients with early-stage HCC, surgical resection or liver transplantation can achieve better survival outcomes. Unfortunately, at diagnosis, less than 15% of patients are eligible for surgery due to multifocality of their disease, inadequate liver function, or early invasion of vascular or biliary structures[7]. The 5-year survival rate for all HCC patients is less than 10%. For the majority of patients with HCC, non-surgical treatment is the only alternative. TACE has long been utilized as a method in palliative treatment for patients with technically unresectable or medically inoperable HCC. Nevertheless, some clinical trials of TACE have shown little survival benefit beyond delaying tumor progression and vascular invasion[8]. The 5-year survival rate for unresectable HCC treated by TACE is only 7%–8%[9]. Therefore, TACE as a monotherapy is a suboptimal treatment for unresectable HCC.

**Table 3:** Multivariate analysis of prognostic predictors for survival rate of hepatocellular carcinoma patients

Parameter	β	SE	Wald	df	Sig	Exp(b)
PTV	-0.339	0.440	0.596	1	0.440	0.712
Child-Pugh grade	2.265	0.646	12.277	1	0.000	9.632
BCLC stage	0.272	0.396	0.472	1	0.492	1.313
TACE times	-0.777	0.152	26.261	1	0.000	0.460
Radiation dose	0.563	0.233	5.825	1	0.016	1.757
HIFU times	-0.618	0.322	3.676	1	0.055	0.539

PTV: planning target volume; BCLC: Barcelona Clinic Liver Cancer; TACE: transcatheter arterial chemoembolization; HIFU: high-intensity focused ultrasound.

(*n* = 120)

Traditionally, HCC has been regarded as a radioresistant tumor. Since 3-DCRT was applied in HCC, it has achieved encouraging outcomes. Seong *et al*.[10] reported a prospective study on 303 HCC patients treated by 3-DCRT, with 88% of patients having tumors >5 cm and 50% having portal vein thromobosis. The 1-year, 3-year, and 5-year survival rates were 45%, 15% and 6%, respectively. However, the 5-year survival rate for HCC treated by 3-DCRT is still undesirable. HIFU is a newly developed noninvasive treatment modality for liver tumors. In a study by Wu *et al*.[11] in which 55 patients with large HCC (with a mean diameter of 8.14 cm) and cirrhosis received HIFU treatment, no major complications were recorded. Completeness of ablation was assessed in 26 patients and the complete ablation rate was 69.2%. The overall survival rates were 61.5% at 12 months and 35.3% at 18 months[12]. To confirm whether TACE combined with 3-DCRT could be an effective and safe modality for HCC patients, we designed this retrospective clinical study. Rationale for this combined therapy was based on the following evidence. First, the efficacy of TACE for patients with unresectable HCC is unsatisfactory due to the disappointing long-term survival rates. Second, microscopic extension of tumor into surrounding liver tissue is not completely treated by TACE since its blood supply may not arise from the hepatic artery. This can lead to frequent tumor recurrences following TACE. In one reported series, the overall cumulative recurrence rate of HCC patients with initial remission following TACE was 23% after 1 year, 55% after 2 years and 67% after 3 years[13]. Third, prior to utilization of 3-DCRT, accuracy in delineation of GTV was limited in many cases due to poorly defined tumor margins of HCC on CT. The deposit of iodized oil after TACE can make the GTV margin sharper, which contributes to a more accurate delineation of GTV. Fourth, the tumor often shrinks due to TACE therapy, which leads to a reduction in irradiation dose to the liver during follow-up radiation therapy. Finally, the chemotherapeutic drug retained in the tumor may augment the anti-tumor efficacy of 3-DCRT. Anti-cancer drugs retained in tumors may have a radiosensitizing effect[14].

In the present study, the combined modality of TACE with 3-DCRT resulted in a satisfactory outcome. Among the 120 patients, PR was observed in 23 patients, SD in 83 patients, and PD in 14 patients. The 1-year, 3-year and 5-year survival rates were 70%, 35% and 15%, respectively. Because of our positive results, we find that TACE combined with 3-DCRT is a useful approach for treatment of HCC. Our study demonstrated that combined therapy of TACE and 3-DCRT was tolerable for the majority of patients with HCC. Acute treatment-related toxicities including hepatic toxicities, gastrointestinal bleeding, and leukopenia were manageable without severe outcomes. The most serious late complication in patients treated by radiation therapy is RILD. In our study, RILD occurred in three cases, which is less than 5% of all patients. RILD is the most serious radiation-induced complication and almost fatal once it has occurred. Since 3-DCRT has allowed the calculation of the effective volume of normal liver irradiated, the risk of RILD could be reduced in the future[15]. With utilization of the active breathing coordinator in 3-DCRT, normal liver irradiation can be reduced even more specifically. Our study has confirmed the efficacy of HIFU for treatment of patients with HCC. However, HIFU is not without complications. Compared with a recent reported series, the complication rate of this series is lower. First- and second-degree skin burn is especially disturbing. Further refinement of the technique, such as artificial ascites and intermittent delivery of acoustic energy to allow skin cooling, has been introduced in our recent practice.

In our study, we determined the following predictors for improved survival of patients with HCC after irradiation by univariate analysis: Child-Pugh grade, BCLC stage, portal vein thormbosis, TACE times and radiation dose. However, Cox proportional regression analysis revealed that only Child-Pugh grade and irradiation dose were independent predictors for overall survival. In the previous literature, portal vein thrombosis was an independent predictive factor for TACE because portal vein thrombosis was associated with acute hepatic failure after TACE[16]. For HCC patients with portal vein thrombosis, percutaneous transhepatic portal vein stenting and TACE combined with 3-DCRT may be an effective treatment modality[17]. But in our study, portal vein thrombosis was not an independent predictive factor because almost all HCC patients with portal vein thrombosis had good liver function (Child-Pugh grade A) except one with Child-Pugh grade B and their main portal vein stenosis was less than 50%. Our study demonstrated that TACE times was not an independent predictive factor as well. This was because different numbers of TACE were performed according to the extent of deposit of iodized oil. TACE would be performed constantly until iodized oil is deposited in the whole tumor. We found that both Child-Pugh grade and radiation dose were independently associated with overall survival rates, which is similar to the previous literature[18]. It is proposed that HCC patients with good liver function (Child-Pugh grade A) and planned radiation dose of > 40 Gy to tumor by conventional fractionation could be an indication for the combined therapy of TACE, 3-DCRT and HIFU. With advances in modern radiotherapy technology such as stereotactic body radiation therapy (SBRT), high doses of radiation to the hepatic tumor can be delivered that can spare the surrounding liver parenchyma and adjacent organs, which may get higher long-term survival rates[19].

In summary, our results demonstrate that the sequential therapy of TACE, 3-DCRT and HIFU is a promising treatment for unresectable HCC. A large-scale, prospective randomized trial should be performed to confirm the utility of this combined therapy.
